# Chemical Constituents of *Caesalpinia decapetala* (Roth) Alston

**DOI:** 10.3390/molecules18011325

**Published:** 2013-01-22

**Authors:** Xiao-Hua Wei, Sheng-Jie Yang, Na Liang, De-Yu Hu, Lin-Hong Jin, Wei Xue, Song Yang

**Affiliations:** Research and Development Center for Fine Chemicals, State Key Laboratory Breeding Base of Green Pesticide and Agricultural Bioengineering, Key Laboratory of Green Pesticide and Agricultural Bioengineering, Ministry of Education, Guizhou University, Guiyang 550025, China; E-Mails: gzuedit@yahoo.com (X.-H.W.); yangsj2003@gmail.com (S.-J.Y.); liangna8703@163.com (N.L.); gzgdjhzx@126.com (D.-Y.H.); linhong_j@126.com (L.-H.J.); shouldww@126.com (W.X.)

**Keywords:** *Caesalpinia decapetala* (Roth) Alston, chemical constituents, structure elucidation, MTT, DPPH

## Abstract

The current study targets the chemical constituents of *Caesalpinia decapetala* (Roth) Alston and investigates the bioactivities of the isolated compounds. Fourteen known compounds were isolated using column chromatography, and structural identification was performed by physical and spectral analyses. The biological activities of the compounds were also evaluated by 3-(4,5-dimethythiazol-2-yl)-2,5-diphenyl tetrazolium bromide (MTT) and 2,2-diphenlyl-1-picrylhydrazyl (DPPH) assays. Emodin (**6**), baicalein (**9**), and apigenin (**12**) displayed antitumor activities against the MGC-803 cell line, while quercetin (**2**), rutin (**5**), baicalein (**9**), and epicatechin (**13**) showed stronger DPPH scavenging activities compared with ascorbic acid. Andrographolide (**1**), quercetin (**2**), bergenin (**4**), rutin (**5**), emodin (**6**), betulin (**7**), baicalein (**9**), polydatin (**10**), salicin (**11**), and apigenin (**12**), were obtained from *C. decapetala* (Roth) Alston for the first time.

## 1. Introduction

*Caesalpinia decapetala* (Roth) Alston is a climbing shrub that belongs to genus *Caesalpinia* of the Fabaceae family. *C. decapetala* (Roth) Alston is widely distributed around the world, but mainly distributed in the southern regions of the Yangtze River in China. The plant is locally known as “Yan wang ci” in Guizhou province, China. The roots of *C. decapetala* (Roth) Alston are used in folk medicine to treat bronchitis, prevent colds, and as an antimalarial agent [1].

Previous chemical investigations on *C. decapetala* (Roth) Alston had revealed that the main chemical components were terpenoids and flavonoids [2,3,4]. Recently, we have systematically investigated the chemical constituents of *C. decapetala* (Roth) Alston and tested the antitumor activities of the compounds to validate the medicinal use of the *C. decapetala* (Roth) Alston. Fourteen known compounds were isolated from the plant, and ten compounds were isolated from *C. decapetala* (Roth) Alston for the first time. In addition, further studies on bioactivities of the isolated compounds were performed to evaluate the *in vitro* anticancer activities of the isolated compounds against the human gastric carcinoma cell line MGC-803. Antioxidant activities were also determined by a 2,2-diphenyl-1-picrylhydrazyl (DPPH) assay. This paper reports the structures and anticancer and antioxidant activities of the isolated compounds. No previous reports have been made on the antitumor, and antioxidant activities of *C. decapetala* (Roth) Alston.

## 2. Results and Discussion

### 2.1. Structural Elucidation of Isolated Compounds

**Figure 1:**
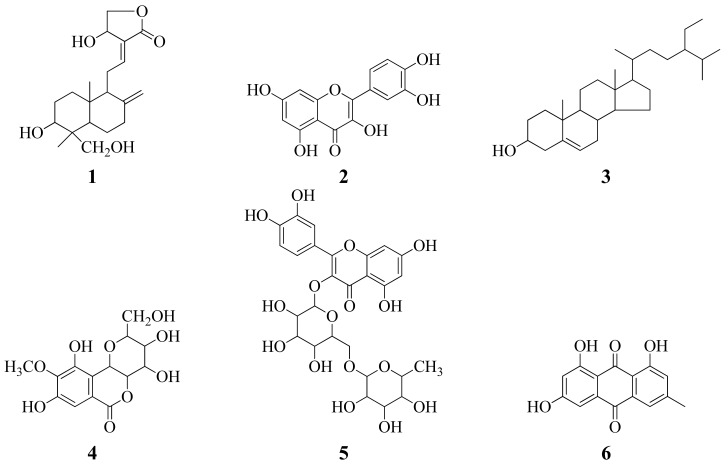

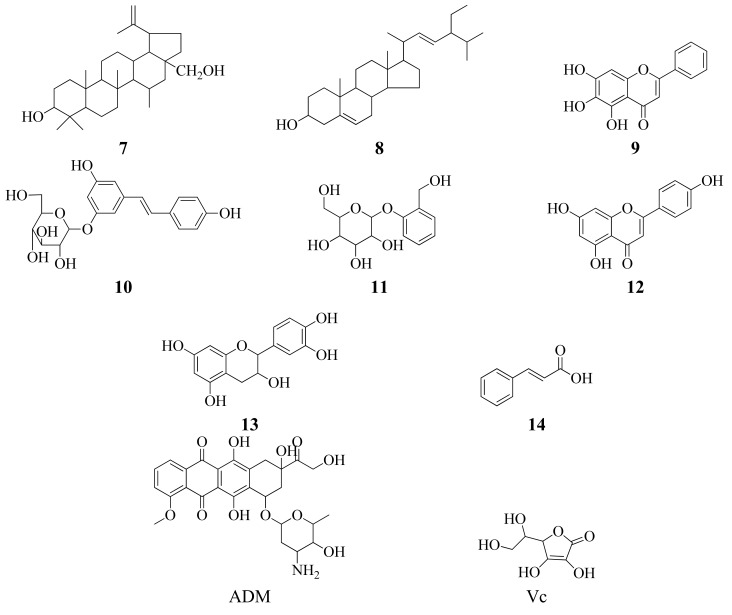
Structures of isolated compounds **1**–**14** and reference substances ADM and Vc.

The isolated compounds were identified via spectroscopic analyses, including ^1^H-NMR and ^13^C-NMR spectroscopy, combined with comparison of measured NMR data with values reported in the literature. The structures of these compounds were shown in Figure 1.

As shown in Figure 1, the compounds isolated from the plant included two terpenoids (compounds **1**, and **7**), five flavones (compounds **2**, **5**, **9**, and **12**–**13**), two sterols (compounds **3**, and **8**), one isocoumarin (compound **4**), one anthraquinone (compound **6**), two polyphenols (compounds **10**–**11**). All compounds, except *β*-sitosterol (**3**), stigmaserol (**8**), epicatechin (**13**) and cinnamic acid (**14**), were isolated from the roots of *C. decapetala* (Roth) Alston for the first time.

### 2.2. Anticancer Activity against MGC-803 Cells *in Vitro*

The antitumor activities of all the isolated compounds were evaluated against MGC-803 cell lines by MTT assay. MTT assay is a widely used method for the detection of cell survival and growth. It is a colorimetric assay that measures the reduction of yellow 3-(4,5-dimethythiazol-2-yl)-2,5-diphenyl tetrazolium bromide (MTT) by mitochondrial succinate dehydrogenase. Adriamycin (ADM) and the compounds were dissolved with DMSO. The negative control cells were treated with culture medium containing 0.1% DMSO, while ADM was used as positive control. The inhibitory percentage of cells was treated with 20 μmol/L of each compound for 72 h. The results are summarized in Table 1. 

As shown in Table 1, among the tested compounds, baicalein (**9**) had the best anticancer activity, with an inhibition rate of 75.7% at a concentration of 20 µmol/L, while apigenin (**12**) had the best anticancer activity with an inhibition rate of 34.1% at a concentration of 5 µmol/L. At a concentration of 20 µmol/L, the relative order of anticancer activity was baicalein (**9**) > apigenin (**12**) > emodin (**6**) > rutin (**5**) > polydatin (**10**) > stigmaserol (**8**), while at a concentration of 5 µmol/L, the relative order of anticancer activity was apigenin (**12**) > emodin (**6**) > polydatin (**10**) > baicalein (**9**) > stigmaserol (**8**) > rutin (**5**). Andrographolide (**1**), bergenin (**4**), salicin (**11**), and epicatechin (**13**) showed no *in vitro* activity against the growth of human gastric carcinoma cell line MGC-803. Apigenin (**12**), baicalein (**9**), and emodin (**6**) generally showed activity against MGC-803 growth, but this activity was lower than that of ADM.

In previous studies, it was found that emodin could increase the Reactive Oxygen Species (ROS) levels of cells and increase the apoptosis-inducing effects, thus enhancing the drug’s cancer cell killing activity [5]. Baicalin can modulate the cell cycle, down-regulate the expression of the bcl-2 and bcl-6, up-regulate the p53, bax and p21 proteins, inhibiting the proliferation of the cell nuclear antigen [6]. Apigenin is widely distributed in fruit and vegetables. It can induce apoptosis in tumor cells, interrupting the signals that reduce the cancer cell proliferation [7].

### 2.3. Antioxidant Activity on DPPH Scavenging Capacities

The DPPH (2,2-diphenyl-1-picrylhydrazyl) assay is a test commonly used to examine the antioxidant activity of some compounds and the tendency of isolated pure compounds to act as hydrogen atom donors. The purple chromogen radical DPPH is reduced by antioxidant compounds to the corresponding pale yellow hydrazine. The antioxidant activity of antioxidant standard was assessed on the basis of radical scavenging effect of the stable DPPH free radical. All the compound dissolved in ethanol, with Vc as positive control. The results are summarized in Table 2.

As shown in Table 2, rutin (**5**) had high DPPH scavenging activity, with the rate of 75.8% at 5 µmol/L, while baicalein (**9**) had high antioxidant activity, with the scavenging rate of 93.4% at 20 µmol/L. The IC_50_ values indicate that quercetin, rutin, baicalein, and epicatechin exhibited high DPPH free radical scavenging activities, which were higher than the positive control Vc. It can be seen that all these compounds contain many phenolic hydroxyl groups, leading to their high DPPH scavenging capacities. The relative order of DPPH scavenging capacity for the tested compounds at a concentration of 5 µmol/L was: rutin (**5**) > baicalein (**9**) > epicatechin (**13**) > quercetin (**2**). At a concentration of 20 µmol/L, the relative order of DPPH scavenging capacity was: baicalein (**9**) > epicatechin (**13**) > quercetin (**2**) > rutin (**5**). Quercetin (**2**), rutin (**5**), baicalein (**9**), and epicatechin (**13**) showed higher antioxidant activities than the reference substance, ascorbic acid (Vc). Quercetin, rutin, baicalein, and epicatechin belong to flavonoids, which have good antioxidant properties due to their special structures [8]. Formation of intramolecular hydrogen bonding or *ortho*-benzoquinone structures through resonance between the semiquinonoid free radical and an *ortho* group can stabilize the semiquinonoid free radical. The number of hydroxyls in a molecule is positively correlated with the antioxidant activity.

## 3. Experimental

### 3.1. General

^1^H-NMR and ^13^C-NMR spectra were obtained on a JEOL-ECX-500 spectrometer using TMS as an internal standard. Column chromatography was performed with silica (200 to 300 mesh, Marine Chemical Industry Factory, Qingdao, China) and D-101 macro reticular resin (100 to 200 mesh, H&E Co., Ltd., Beijing, China). The chemical reagents used were all of analytical grade.

### 3.2. Plant Materials

*C. decapetala* (Roth) Alston roots were collected in October 2010 from Anshun, Guizhou, China and authenticated by Professor Long Qing De of Guiyang Medical College. A voucher specimen of the collection was deposited at the Center for Research and Development of Fine Chemicals of Guizhou University.

### 3.3. Extraction and Isolation of the Compounds

**Figure 2:**
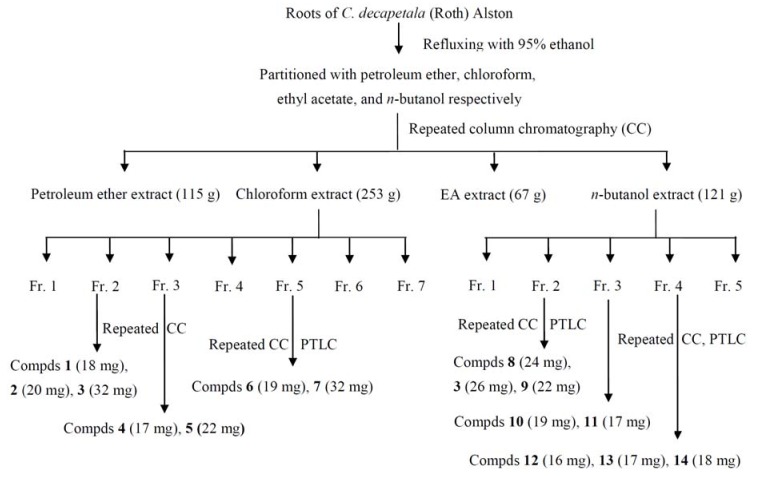
Extraction and column chromatography separation of *C. decapetala* (Roth) Alston roots.

Air-dried *C. decapetala* (Roth) Alston roots (15 kg) were extracted three times with ethanol (50 L) under reflux (2 h each time). The combined EtOH extracts were evaporated to dryness to yield the crude extract (600 g). The extract was then suspended in water and then extracted successively with petroleum ether (10 L × 3 times), ethyl acetate (10 L × 3 times) and *n*-BuOH (10 L × 3 times) respectively, to yield the petroleum ether extract (115 g), chloroform extract (253 g), ethyl acetate extract (67 g), and *n*-BuOH extract (121 g). The organic chloroform extract (253 g) was subjected to repeated column chromatography on silica gel (200–300 mesh) eluted with a gradient of petroleum ether-EtoAc to afford seven fractions, namely, Fractions 1–7. Fractions 2, 3, and 5 of the chloroform extract were further subjected to silica gel column chromatography, with the petroleum ether-ethyl acetate (1:0–0:1, v/v) gradient elution, purified by normal-phase preparative TLC, and recrystallized to afford compounds **1** to **7**. The *n*-butanol extract was separated and purified into five fractions by D-101 macro reticular resin gradient elution with ethanol/water. Fractions 2, 3, and 4 of the *n*-butanol extract were further subjected to repeated silica gel column chromatography, with an ethyl acetate-methanol (1:0–0:1, v/v) gradient eluent, purified by normal-phase preparative TLC, and recrystallized to afford compounds **8** to **14**. The separation procedures are summarized in Figure 2.

### 3.4. Spectroscopic Data

*Andrographolide* (**1**): C_20_H_30_O_5_, white powder, m.p. 230–232 °C. ESI-MS (Positive mode) *m/z * 373.3 [M+Na]^+^. ^1^H-NMR (CD_3_OD, 500 MHz), *δ*: 0.71 (3H, s, CH_3_-20), 1.23 (3H, s, CH_3_-18), 1.81 (1H, s, H-5), 2.00 (1H, s, H-11), 2.41 (1H, d, *J* = 12.6 Hz, H-11), 2.58 (1H, s, H-9), 4.16 (1H, d, *J* = 10.9 Hz, H-19), 4.21 (1H, d, *J* = 10.3 Hz, H-19), 4.40 (1H, brs, H-15), 4.46 (1H, brs, H-3), 4.54 (1H, brs, H-15), 4.63 (1H, s, H-14), 4.88 (1H, s, H-17), 4.97 (1H, s, H-17), 6.90 (1H, s, H-12); ^13^C-NMR (CD_3_OD, 125 MHz) *δ*: 37.0 (C-1), 27.8 (C-2), 79.9 (C-3), 42.4 (C-4), 55.2 (C-5), 23.8 (C-6), 37.7 (C-7), 148.8 (C-8), 56.1 (C-9), 38.8 (C-10), 24.7 (C-11), 146.8 (C-12), 128.1 (C-13), 65.5 (C-14), 74.9 (C-15), 171.4 (C-16), 108.7 (C-17), 22.6 (C-18), 64.0 (C-19), 15.0 (C-20). The above data were identical to those in the literature data [9].

*Quercetin* (**2**): C_15_H_10_O_7_, yellow powder, m.p. 314–316 °C. ESI-MS (Positive mode) *m/z* 301.3 [M−H]^+^. ^1^H-NMR (CD_3_OD, 500 MHz) *δ*: 6.16 (1H, d, *J* = 2.3 Hz, H-6), 6.37 (1H, d, *J* = 2.3 Hz, H-8), 6.86 (1H, d, *J* = 8.5 Hz, H-5'), 7.61 (1H, dd, *J* = 1.7 Hz, 2.3 Hz, H-6'), 7.71 (1H, d, *J* = 1.7 Hz, H-2'); ^13^C-NMR (CD_3_OD, 125 MHz) *δ*: 146.7 (C-2), 135.9 (C-3), 176.0 (C-4), 161.2 (C-5), 97.9 (C-6), 164.3 (C-7), 93.1 (C-8), 156.9 (C-9), 103.2 (C-10), 120.3 (C-1'), 114.6 (C-2'), 144.9 (C-3'), 114.9 (C-5'), 122.8 (C-6'). The data were equal to those of literature data [10].

*β-Sitosterol* (**3**): C_29_H_50_O, white crystals, m.p. 138–139 °C. ESI-MS (Positive mode) *m/z* 413.5 [M−H]^+^, 397.5 [M-OH]^+^. ^1^H-NMR (CDCl_3_, 500 MHz) *δ*: 5.35 (1H, d, *J* = 5.1 Hz, H-6), 3.53 (1H, m, H-3), 1.01 (3H, s, CH_3_-19), 0.92 (3H, d, *J* = 6.3 Hz, CH_3_-21), 0.84 (3H, d, *J* = 1.7 Hz, CH_3_-29), 0.82 (3H, d, *J* = 4.5 Hz, CH_3_-26), 0.81 (3H, s, CH_3_-27), 0.68 (3H, s, CH_3_-18); ^13^C-NMR (CDCl_3_, 125 MHz) *δ*: 37.3 (C-1), 32.0 (C-2, C-8), 71.9 (C-3), 42.4 (C-4), 140.8 (C-5), 121.8 (C-6), 31.8 (C-7), 50.2 (C-9), 36.6 (C-10), 21.2 (C-11), 39.9 (C-12), 42.3 (C-13), 56.8 (C-14), 24.4 (C-15), 28.3 (C-16), 56.1 (C-17), 11.9 (C-18), 18.9 (C-19), 36.2 (C-20), 19.1 (C-21), 34.0 (C-22), 26.1 (C-23), 45.9 (C-24), 29.2 (C-25), 19.9 (C-26), 19.5 (C-27), 23.1 (C-28), 12.1 (C-29). These data were consistent with those reported in the literature data [11].

*Bergenin* (**4**): C_14_H_16_O_9_, white powder, m.p. 236–238 °C. ESI-MS (Positive mode) *m/z* 349.3 [M+Na]^+^. ^1^H-NMR (CD_3_OD) *δ*: 3.20 (1H, dd, *J* = 1.7 Hz, 1.7 Hz, H-12), 3.33 (1H, t, *J* = 9.1 Hz, 9.2 Hz, H-14_a_), 3.58 (1H, m, H-13), 3.70 (1H, t, *J* = 9.1 Hz, 9.1 Hz, H-11), 3.79 (3H, s, OCH_3_), 3.91 (1H, s, H-14_b_), 3.94 (1H, dd, *J* = 3.4 Hz, 9.7 Hz, H-3), 4.84 (1H, d, *J* = 10.3 Hz, H-4), 6.97 (1H, s, H-9); ^13^C-NMR (CD_3_OD) *δ*: 164.5 (C-1), 80.1 (C-3), 72.9 (C-4), 118.1 (C-5), 151.0 (C-6), 148.1 (C-7), 140.9 (C-8), 109.7 (C-9), 116.0 (C-10), 74.3 (C-11), 70.5 (C-12), 81.7 (C-13), 61.3 (C-14), 59.6 (C-15). The above data were consistent with the literature data [12].

*Rutin* (**5**): C_27_H_30_O_16_, yellow powder, m.p. 176–178 °C. ESI-MS (Positive mode) *m/z* 633.3 [M+Na]^+^. ^1^H-NMR (CD_3_OD) *δ*: 1.10 (3H, d, *J* = 6.3 Hz, CH_3_-6'''), 3.79 (2H, d, *J* = 9.7 Hz, H-6''), 4.50 (1H, d, *J* = 1.1 Hz, H-1'''), 5.09 (1H, d, *J* = 8.0 Hz, H-1''), 6.19 (1H, d, *J* = 1.7 Hz, H-6), 6.38 (1H, d, *J* = 2.3 Hz, H-8), 6.86 (1H, d, *J* = 8.0 Hz, H-5'), 7.61 (1H, dd, *J* = 2.3 Hz, 2.3 Hz, H-6'), 7.65 (1H, d, *J* = 2.3 Hz, H-2'); ^13^C-NMR (CD_3_OD) *δ*: 157.2 (C-2), 134.3 (C-3), 178.1 (C-4), 161.6 (C-5), 98.6 (C-6), 164.8 (C-7), 93.5 (C-8), 158.0 (C-9), 104.3 (C-10), 121.8 (C-1'), 114.7 (C-2'), 144.5 (C-3'), 148.5 (C-4'), 116.3 (C-5'), 122.2 (C-6'), 103.4 (C-1''), 74.4 (C-2''), 76.8 (C-3''), 70.1 (C-4''), 75.9 (C-5''), 67.2 (C-6''), 101.1 (C-1'''), 70.8 (C-2'''), 70.9 (C-3'''), 72.6 (C-4'''), 68.4 (C-5'''), 16.5 (C-6'''). These data were in accordance with those reported in the literature data [13].

*Emodin* (**6**): C_15_H_10_O_5_, red powder, m.p. 256–257 °C. ESI-MS (Positive mode) *m/z* 271.2 [M+H]^+^. ^1^H-NMR (CD_3_OD) *δ*: 2.40 (3H, s, CH_3_-6), 6.51 (1H, d, *J* = 2.8 Hz, H-2), 7.03 (1H, s, H-4), 7.12 (1H, d, *J* = 2.3 Hz, H-7), 7.49 (1H, s, H-5); ^13^C-NMR (CD_3_OD) *δ*: 20.7 (CH_3_), 165.2 (C-1), 107.7 (C-2), 166.0 (C-3), 108.8 (C-4), 120.4 (C-5), 148.4 (C-6), 123.9 (C-7), 162.2 (C-8), 190.6 (C-9), 181.9 (C-10), 135.5 (C-4_a_), 113.5 (C-9_a_), 109.1 (C-8_a_), 133.3 (C-10_a_). These data were identical to those in the literature data [14].

*Betulin* (**7**): C_30_H_50_O_2_, white powder, m.p. 256–258 °C. ESI-MS (Positive mode) *m/z* 443.5 [M+H]^+^. ^1^H-NMR (CDCl_3_) *δ*: 0.75, 0.81, 0.96, 0.97, 1.04, 1.67 (each 3H, s, CH_3_×6), 3.18 (1H, dd, *J* = 4.6 Hz, 4.6 Hz, H-3), 3.32 (1H, d, *J* = 10.9 Hz, H-28_a_), 3.79 (1H, d, *J* = 10.8 Hz, H-28_b_), 4.57 (1H, s, H-29_a_), 4.67 (1H, s, H-29_b_); ^13^C-NMR (CDCl_3_) *δ*: 38.8 (C-1), 27.5 (C-2), 79.1 (C-3), 38.9 (C-4), 55.4 (C-5), 18.4 (C-6), 34.3 (C-7), 41.0 (C-8), 50.5 (C-9), 37.4 (C-10), 20.9 (C-11), 25.3 (C-12), 37.2 (C-13), 42.8 (C-14), 27.1 (C-15), 29.2 (C-16), 47.9 (C-17, C-18), 48.8 (C-19), 150.6 (C-20), 29.8 (C-21), 34.1 (C-22), 28.1 (C-23), 15.5 (C-24), 16.2 (C-25), 16.1 (C-26), 14.8 (C-27), 60.6 (C-28), 19.2 (C-29), 109.8 (C-30). These data were consistent with those previously reported in the literature data [15].

*Stigmaserol* (**8**): C_29_H_48_O, white powder, m.p. 140–142 °C. ESI-MS (Positive mode) *m/z* 413.4 [M+H]^+^, 395.5 [M-OH]^+^. ^1^H-NMR (CDCl_3_) *δ*: 0.69 (3H, s, CH_3_-13), 0.80 (3H, t, *J* = 7.4 Hz, 7.4 Hz, CH_3_-29), 0.83 (3H, s, CH_3_-26), 0.85 (3H, s, CH_3_-27), 1.00 (3H, s, CH_3_-19), 1.02 (3H, s, CH_3_-21), 5.01 (1H, dd, *J* = 8.6 Hz, 8.6 Hz, H-23), 5.14 (1H, dd, *J* = 8.6 Hz, 8.6 Hz, H-22), 5.34 (1H, d, *J* = 5.1 Hz, H-6); ^13^C-NMR (CDCl_3_) *δ*: 37.3 (C-1), 31.8 (C-2), 71.9 (C-3), 42.4 (C-4), 140.8 (C-5), 121.8 (C-6), 31.8 (C-7), 32.0 (C-8), 50.2 (C-9), 36.6 (C-10), 21.2 (C-11), 39.8 (C-12), 42.3 (C-13), 57.0 (C-14), 24.5 (C-15), 29.0 (C-16), 56.0 (C-17), 12.4 (C-18), 19.5 (C-19), 40.6 (C-20), 21.2 (C-21), 138.4 (C-22), 129.3 (C-23), 51.3 (C-24), 32.0 (C-25), 19.1 (C-26), 21.3 (C-27), 25.5 (C-28), 12.1 (C-29). The spectral data were in accordance the reported data in the literature data [16].

*Baicalein* (**9**): C_15_H_10_O_5_, yellow needle crystals, m.p. 264–265 °C. ESI-MS (Positive mode) *m/z* 293.2 [M+Na]^+^, 271.2 [M+H]^+^. ^1^H-NMR (CD_3_OD) *δ*: 6.59 (1H, s, H-8), 6.70 (1H, s, H-3), 7.54 (3H, m, H-3', H-4', H-5'), 7.95 (2H, dd, *J* = 1.7 Hz, 1.1 Hz, H-2', H-6'); ^13^C-NMR (CD_3_OD) *δ*: 164.3 (C-2), 104.0 (C-3), 182.9 (C-4), 146.6 (C-5), 129.4 (C-6), 153.6 (C-7), 93.7 (C-8), 150.8 (C-9), 104.5 (C-10), 131.4 (C-1'), 126.0 (C-2', C-6'), 128.9 (C-3', C-5'), 131.6 (C-4'). These data were identical to those in the literature data [17].

*Polydatin* (**10**): C_20_H_22_O_8_, white crystals, m.p. 224–226 °C. ESI-MS (Positive mode) *m/z* 413.2 [M+Na]^+^. ^1^H-NMR (CD_3_OD) *δ*: 3.35 (1H, s, H-4''), 3.36 (1H, s, H-3''), 3.38 (1H, s, H-5''), 3.41 (1H, s, H-2''), 3.44 (1H, t, *J* = 6.3 Hz, 7.4 Hz, H-6''_a_), 3.69 (1H, dd, *J* = 5.7 Hz, 5.7 Hz, H-6''_b_), 3.91 (1H, dd, *J* = 1.7 Hz, 1.7 Hz, H-1''), 6.43 (1H, t, *J* = 1.7 Hz, 2.3 Hz, H-4), 6.59 (1H, s, H-6), 6.75 (2H, t, *J* = 10.1 Hz, 8.6 Hz, H-3', H-5'), 6.83 (1H, d, *J* = 16.6 Hz, H-*α*), 7.00 (1H, d, *J* = 16.6 Hz, H-*β*), 7.35 (2H, d, *J* = 8.6 Hz, H-2', H-6'); ^13^C-NMR (CD_3_OD) *δ*: 140.1 (C-1), 102.7 (C-2), 159.1 (C-3), 105.6 (C-4), 158.3 (C-5), 107.0 (C-6), 128.6 (C-*β*), 125.3 (C-*α*), 129.0 (C-1'), 127.6 (C-2', C-6'), 115.1 (C-3', C-5'), 101.1 (C-1''), 73.6 (C-2''), 76.7 (C-3''), 70.1 (C-4''), 76.9 (C-5''), 61.2 (C-6''). These data were in accordance with those reported previously in the literature data [18].

*Salicin* (**11**): C_13_H_18_O_7_, white crystals, m.p. 190–192 °C. ESI-MS (Positive mode) *m/z* 309.2 [M+Na]^+^. ^1^H-NMR (CD_3_OD) *δ*: 4.55 (1H, d, *J* = 13.1 Hz, H-7), 4.76 (1H, d, *J* = 12.6 Hz, H-7), 7.01 (1H, t, *J* = 7.4 Hz, 7.4 Hz, H-4), 7.19 (1H, d, *J* = 8.6 Hz, H-6), 7.24 (1H, t, *J* = 6.8 Hz, 8.6 Hz, H-5), 7.32 (1H, d, *J* = 6.9 Hz, H-3); ^13^C-NMR (CD_3_OD) *δ*: 155.8 (C-1), 130.8 (C-2), 128.6 (C-3), 122.4 (C-4), 128.5 (C-5), 115.7 (C-6), 59.6 (C-7), 102.0 (C-1'), 73.7 (C-2'), 76.9 (C-3'), 70.0 (C-4'), 76.7 (C-5'), 61.2 (C-6'). These data were identical to those in the literature data [19].

*Apigenin* (**12**): C_15_H_10_O_5_, yellow powder, m.p. 347–349 °C. ESI-MS (Positive mode) *m/z* 293.2 [M+Na]^+^, 271.2 [M+H]^+^. ^1^H-NMR (CDCl_3_) *δ*: 6.27 (1H, d, *J* = 1.7 Hz, H-6), 6.46 (1H, d, *J* = 1.7 Hz, H-8), 6.55 (1H, d, *J* = 8.0 Hz, H-3), 6.95 (1H, d, *J* = 8.6 Hz, H-3', H-5'), 7.82 (2H, d, *J* = 8.6 Hz, H-2', H-6'); ^13^C-NMR (CDCl_3_) *δ*: 165.0 (C-2), 102.9 (C-3), 182.7 (C-4), 161.6 (C-5), 99.2 (C-6), 164.4 (C-7), 94.2 (C-8), 158.1 (C-9), 104.4 (C-10), 122.1 (C-1'), 128.3 (C-2', C-6'), 115.9 (C-3', C-5'), 161.1 (C-4'). The spectral data were in accordance with the known compound apigenin in the literature data [20].

*Epicatechin* (**13**): C_15_H_14_O_6_, white crystals, m.p. 224–226 °C. ESI-MS (Positive mode) *m/z* 313.2 [M+Na]^+^, 291.2 [M+H]^+^. ^1^H-NMR (CDCl_3_) *δ*: 2.81 (1H, dd, *J* = 2.8 Hz, 2.9 Hz, H-4_b_), 2.90 (1H, dd, *J* = 4.0 Hz, 4.5 Hz, H-4_a_), 4.22 (1H, s, H-3), 4.86 (1H, s, H-2), 5.98 (1H, d, *J* = 2.2 Hz, H-6), 6.00 (1H, d, *J* = 2.3 Hz, H-8), 6.82 (2H, s, H-5', H-6'), 7.00 (1H, s, H-2'); ^13^C-NMR (CDCl_3_) *δ*: 78.5 (C-2), 66.3 (C-3), 28.0 (C-4), 156.2 (C-5), 95.5 (C-6), 156.6 (C-7), 95.0 (C-8), 155.9 (C-9), 97.8 (C-10), 130.6 (C-1'), 113.9 (C-2'), 144.4 (C-3'), 144.6 (C-4'), 118.3 (C-5'), 115.0 (C-6'). These data were consistent with those reported in the literature data [21].

*Cinnamic acid* (**14**): C_9_H_8_O_2_, white crystals, m.p. 131–133 °C. ESI-MS (Positive mode) *m/z* 171.1 [M+Na]^+^, 131.2 [M-OH]^+^. ^1^H-NMR (CDCl_3_) *δ*: 6.46 (1H, d, *J *= 16.0 Hz, H-*β*), 7.41 (3H, t, H-3, H-4, H-5), 7.56 (2H, dd, *J *= 3.4 Hz, 1.7 Hz, H-2, H-6), 7.80 (1H, d, *J *= 16.0 Hz, H-*γ*); ^13^C-NMR (CDCl_3_) *δ*: 134.1 (C-1), 128.5 (C-2, C-6), 129.1 (C-3, C-5), 130.9 (C-4), 172.5 (C-*α*), 117.4 (C-*β*), 147.2 (C-*γ*). These data were identical to those in the literature data [22].

### 3.5. Cell Lines and Culture

MGC-803 from stomach cancer was obtained from the Institute of Biochemistry and Cell Biology of the China Academy of Science (Shanghai, China). MGC-803 cells were maintained in RPMI 1640 medium and supplemented with 10% heat-inactivated fetal bovine serum in a humidified atmosphere of 5% CO_2_ at 37 °C.

### 3.6. MTT Assays

All tested compounds were separately dissolved in DMSO and subsequently diluted in the culture medium before treatment of the cells. Tested cells were plated in 96-well plates at a density of 2 × 10^4^ cells/well/100 µL of the proper culture medium and treated with the compounds at different concentrations for 72 h. In parallel, cells treated with 0.1% DMSO and ADM served as the negative and positive controls, respectively. Finally, 100 µL of MTT was added and the cells were incubated for 4 h. The MTT-formazan formed from metabolically viable cells was dissolved in 100 µL of SDS for 12 h. The absorbance, which is directly proportional to the number of living cells in the culture, was then measured at 595 nm with a micro-plate reader (BIO-RAD, Model 680, Philadelphia, PA, USA), which is directly proportional to the number of living cells in culture [23]. Cytotoxicity was calculated using the following formula. The IC_50_ value, for the tested compounds by determining the concentration needed to inhibit half of the maximum biological response of the tumor cell proliferation, were calculated from the results:

$$\text{\% Cytotoxicity}=\frac{\left(Control(Abs)-Blank(Abs))-(Test(Abs)-Blank(Abs)\right)}{\left(Control(Abs)-Blank(Abs)\right)}\times 100$$

### 3.7. DPPH Assays

DPPH scavenging capacity was measured using the method described by Sun Tao *et al*. [24] with slight modifications. Briefly, a 20 µmol/L solution of DPPH radical solution in ethanol was prepared and 2 mL of this solution was added to the antioxidant solutions (2 mL) in DMSO at different concentrations. The mixture was shaken vigorously, after a 30 min incubation period at 25 °C in the dark, the absorbance of the resulting solutions was measured at 517 nm with ascorbic acid as the reference substance. The DPPH radical scavenging effect was calculated using follow equation:

$$\text{\% Scavenging activity}=(1-\frac{A2-A1}{A0})\times 100$$

where A_0_ is the absorbance of DPPH without the sample liquid, A_1_ is the absorbance of the sample liquid without DPPH, and A_2_ is the absorbance of the reactive fluid. The IC_50_ value, for the tested compounds by determining to be the effective concentration at which DPPH radical was scavenged by 50%, was calculated from the results.

## 4. Conclusions

In this study, fourteen compounds were isolated from* Caesalpinia decapetala* (Roth) Alston, identified as Andrographolide (**1**), quercetin (**2**), *β*-sitosterol (**3**), bergenin (**4**), rutin (**5**), emodin (**6**), betulin (**7**), stigmaserol (**8**), baicalein (**9**), polydatin (**10**), salicin (**11**), apigenin (**12**), epicatechin (**13**) and cinnamic acid (**14**). Compounds **1-2**, **4-7**, and **9-12** were obtained from the plant for the first time. In addition, the antitumor and antioxidant activities of these compounds were evaluated. The results revealed that emodin (6), baicalein (9), and apigenin (12) were shown significant antitumor activities against MGC-803 cell lines, with IC_50_ values of 15.6, 16.3, and 13.2 µmol/L, respectively, and most of the flavonoids displayed significant DPPH scavenging capacities, with baicalein (**9**), epicatechin (**13**), quercetin (**2**), and rutin (**5**) showing stronger activities than ascorbic acid. The antioxidant activity of the flavonoids may be related to their structures. Antioxidant activity is most significant in flavonoids, and the antioxidant activities of flavonoids are realized by inactivation of free radicals. Because flavonoids are potential hydrogen donors, the isolated flavonoids inhibit the production of free radicals, increasing the potential antioxidant activity. The flavonoid-rich plant *C. decapetala* (Roth) Alston, therefore exhibits biological activities, in particular, antioxidant activity. The chemical composition and biological activity of this traditional medicinal plant deserve further study.

## Figures and Tables

**Table 1:** Antitumor activities of the tested compounds from *Caesalpinia decapetala* (Roth) Alston on the growth of human gastric carcinoma cell line MGC-803 *in vitro*.

Compound	Inhibitory rate (%, mean ± SD) ^a^		IC_50_ (µmol/L, mean ± SD)
	5 µmol/L	20 µmol/L	
Andrographolide (**1**)	NT ^b^	NT	NT
Bergenin (**4**)	NT	NT	NT
Rutin (**5**)	2.4 ± 14.1	11.2 ± 7.8	NT
Emodin (**6**)	29.5 ± 6.4	62.7 ± 3.6	15.6 ± 0.42
Stigmaserol (**8**)	3.1 ± 6.8	8.6 ± 7.2	NT
Baicalein (**9**)	3.4 ± 6.0	75.7 ± 2.0	16.3 ± 0.51
Polydatin (**10**)	17.9 ± 5.1	9.8 ± 6.7	NT
Salicin (**11**)	NT	NT	NT
Apigenin (**12**)	34.1 ± 12.0	67.1 ± 7.1	13.2 ± 0.32
Epicatechin (**13**)	NT	NT	NT
ADM ^c^	63.7 ± 1.8	94.4 ± 1.0	0.4 ± 0.10

Note: ^a^ Inhibitory percentage of cells treated with 5 µmol/L and 20 µmol/L of each compound for 72 h and SD = standard deviation; ^b^ NT indicate not available because of low activity; ^c^ The standard compound used for comparison of activities.

**Table 2:** Antioxidant activities of the tested compounds from *Caesalpinia decapetala* (Roth) Alston on DPPH scavenging capacities.

Compound	Scavenging rate (%, mean ± SD) ^a^		IC_50_ (µmol/L, mean ± SD)
	5 µmol/L	20 µmol/L	
Andrographolide (**1**)	NT ^b^	13.5 ± 1.4	NT
Quercetin (**2**)	40.4 ± 0.7	82.7 ± 1.3	16.3 ± 0.52
Bergenin (**4**)	NT	15.5 ± 0.9	NT
rutin (**5**)	75.8 ± 1.3	80.9 ± 0.7	14.2 ± 0.34
Emodin (**6**)	3.6 ± 0.3	20.2 ± 1.5	NT
Betulin (**7**)	2.5 ± 0.7	5.8 ± 1.4	NT
Stigmaserol (**8**)	NT	NT	NT
baicalein (**9**)	64.7 ± 1.1	93.4 ± 0.5	12.7 ± 0.25
Polydatin (**10**)	8.7 ± 1.0	36.1 ± 0.6	NT
salicin (**11**)	0.3 ± 0.5	6.0 ± 0.3	NT
apigenin (**12**)	0.2 ± 0.3	6.0 ± 0.6	NT
epicatechin (**13**)	59.2 ± 0.5	86.7 ± 0.6	15.5 ± 0.42
Vc ^c^	23.4 ± 0.7	74.7 ± 0.3	18.2 ± 0.3

Note: ^a^ Inhibitory percentage of DPPH treated with 5 µmol/L and 20 µmol/L and SD = standard deviation; ^b^ NT indicate not available because of low activity; ^c^ The standard compound used for comparison of activities.
